# Nanoelectromechanical Sensors Based on Suspended 2D Materials

**DOI:** 10.34133/2020/8748602

**Published:** 2020-07-20

**Authors:** Max C. Lemme, Stefan Wagner, Kangho Lee, Xuge Fan, Gerard J. Verbiest, Sebastian Wittmann, Sebastian Lukas, Robin J. Dolleman, Frank Niklaus, Herre S. J. van der Zant, Georg S. Duesberg, Peter G. Steeneken

**Affiliations:** ^1^Chair of Electronic Devices, RWTH Aachen University, Otto-Blumenthal-Str. 2, 52074 Aachen, Germany; ^2^AMO GmbH, Advanced Microelectronic Center Aachen (AMICA), Otto-Blumenthal-Str. 25, 52074 Aachen, Germany; ^3^Institute of Physics, Faculty of Electrical Engineering and Information Technology, Universität der Bundeswehr München, Werner-Heisenberg-Weg 39, 85577 Neubiberg, Germany; ^4^Division of Micro and Nanosystems, KTH Royal Institute of Technology, Malvinas Väg 10, 10044 Stockholm, Sweden; ^5^Department of Precision and Microsystems Engineering, Delft University of Technology, Mekelweg 2, 2628 CD Delft, Netherlands; ^6^Infineon Technologies AG, Wernerwerkstraße 2, 93049 Regensburg, Germany; ^7^2nd Institute of Physics, RWTH Aachen University, Otto-Blumenthal-Str., 52074 Aachen, Germany; ^8^Kavli Institute of Nanoscience, Delft University of Technology, Lorentzweg 1, 2628 CJ Delft, Netherlands

## Abstract

The unique properties and atomic thickness of two-dimensional (2D) materials enable smaller and better nanoelectromechanical sensors with novel functionalities. During the last decade, many studies have successfully shown the feasibility of using suspended membranes of 2D materials in pressure sensors, microphones, accelerometers, and mass and gas sensors. In this review, we explain the different sensing concepts and give an overview of the relevant material properties, fabrication routes, and device operation principles. Finally, we discuss sensor readout and integration methods and provide comparisons against the state of the art to show both the challenges and promises of 2D material-based nanoelectromechanical sensing.

## 1. Introduction

Two-dimensional (2D) materials have excellent material properties for sensor applications due to their large surface-to-volume ratio and unique electrical, mechanical, and optical properties [1, 2]. More recently, the potential of 2D materials for sensing has been further extended by freely suspending 2D materials to form atomically thin membranes, ribbons, or beams [3–6]. These types of suspended 2D material structures enable a new class of 2D suspended NEMS sensors, which is the focus of the present review. Suspending 2D materials eliminates substrate interactions, increases their thermal isolation, and gives them freedom of motion, which opens a whole range of mechanical sensing modalities. In fact, many of the current micro- and nanoelectromechanical system (MEMS and NEMS) devices can be realized using suspended 2D materials, offering smaller dimensions, higher sensitivity, and novel functionalities compared to their silicon-based MEMS and NEMS counterparts. This is because the performance and sensitivity of NEMS sensors often depend critically on the thickness of the suspended membrane or beam, which can reach its ultimate thinness when using suspended 2D materials. Moreover, new types of sensors can be enabled by exploiting the unique properties of 2D materials. Sensors in which the nanomechanical and/or electrical response of suspended 2D materials is used to sense environmental parameters can be classified as 2D material NEMS sensors. Such 2D NEMS sensors therefore have the potential to provide novel and/or better solutions for applications such as the Internet of Things (IoT) and autonomous mobility, which are expected to drive the demand for integrated and high-performance sensors for years to come.

Early studies investigated the application of graphene in NEMS as resonant structures [7], which provide ultimate sensitivity for mass detection down to the hydrogen atom limit [8]. An overview of graphene-based nanoelectromechanical resonators was provided in a 2013 review paper [9], and the utilization of graphene and carbon nanotubes in NEMS was briefly summarized by Zang et al. [10]. However, it has recently become clear that graphene has potential for enabling a much wider range of NEMS sensors, with transition metal dichalcogenide (TMD) and 2D semiconductor materials also emerging in this application space [6, 11, 12].

In this work, we present a review of 2D material NEMS sensors based on suspended graphene and related 2D materials operating in vacuum or gaseous environments. We discuss the relevant material properties, describe key fabrication technologies, and evaluate the potential for Complementary Metal Oxide Semiconductor (CMOS) integration of 2D material NEMS sensors, specifically focusing on those topics relevant for these sensors that are not covered by previous reviews [13–15]. We present suitable transduction mechanisms that are of particular relevance to NEMS sensors and finally review the state of the art in 2D membrane-based NEMS sensor applications, discussing pressure sensors, accelerometers, oscillators, resonant mass sensors, gas sensors, Hall effect sensors, and bolometers. This latter part of the paper is organized by application, not by material.

## 2. Material Properties of Suspended 2D Materials

In designing sensors and deciding on how to fabricate them, it is important to select a suitable 2D material. For that purpose, we discuss here the material properties that are relevant for nanoelectromechanical sensing. In fact, not all 2D materials are suitable to form suspended structures. As for graphene, many of its material properties are beneficial for forming freely suspended membranes, beams, and ribbons, including chemical stability at atmospheric conditions, excellent mechanical robustness, stretchability of up to about 20% [16], a Young's modulus of 1 TPa [17], intrinsic strength of 130 GPa [17], room-temperature electron mobility of 2.5 × 10^5^ cm^2^/Vs [18], excellent transparency, uniform optical absorption of ≈2.3% in a wide wavelength range [19], impermeability to gases [20, 21] (except hydrogen [22]), and the ability to sustain extremely high current densities [23]. Because graphene shows very strong adhesion to SiO_2_ surfaces [24], it can be suspended in one atom layer thick membranes that are mechanically stable [25] and can be readily chemically functionalized [26]. However, it is important to point out that some of the extreme properties have been measured only in mechanically exfoliated, high-quality graphene samples that do not contain grain boundaries [27] or for graphene on specific substrates such as hexagonal boron nitride [18, 28].

Beyond graphene, other 2D materials also show promising properties for the use as membrane sensors, such as their relatively high in-plane stiffness and strength [29]. For instance, Young's moduli of monolayer h-BN, MoS_2_, WS_2_, MoSe_2_, and multilayer WSe_2_ are reported to be 865 GPa, 270 GPa, 272 GPa, 177 GPa, and 167 GPa, respectively [29], in line with theoretical predictions [30]. Furthermore, the intrinsic strength of h-BN and MoS_2_, two of the most studied 2D materials beyond graphene, is reported to be ~70.5 GPa and~22 GPa, with fracture strains of 6-11% and 17%, respectively [29], comparable to graphene. Hexagonal BN is an insulator that is used as a substrate and as encapsulation material for graphene and other 2D materials to improve their electronic transport properties [28] and mechanical stability. The piezoresistive gauge factors of monolayer MoS_2_ and bilayer MoS_2_ and PtSe_2_ have been reported to be about −148 ± 19, −224 ± 19, and −84 ± 23, respectively [6, 31], which are up to two orders of magnitude higher than commonly reported values in graphene with gauge factors (GF) between 2 and 6 [25, 32–35]. Therefore, compared to graphene, transition metal dichalcogenides (TMDs) offer piezoresistive readout of NEMS with much higher responsivity. Other 2D TMDs such as WS_2_, MoSe_2_, and WSe_2_ are also predicted to have much higher piezoresistive gauge factors than graphene [36, 37], emphasizing the potential of TMD-based piezoresistive membrane sensors.  Table 1 compares the 2D material properties that are most relevant and interesting for applications based on suspended membranes, such as Young's modulus, piezoresistive gauge factor, and optical bandgap.

The values in Table 1 are extracted from measurements at room temperature under application relevant conditions. Some properties like charge carrier mobility values have only partly been investigated for the suspended 2D materials. The terms “suspended” and “supported” therefore indicate how the value was obtained. In general, due to differences in fabrication and characterization procedures, large variations in the different material properties are found in literature, which leaves many open questions for NEMS device functionality. In addition, built-in stress in suspended 2D materials is generally large and difficult to control, while having a tangible influence on the static and dynamic characteristics of 2D material NEMS [72]. Built-in stress in fully clamped graphene membranes can reach 10^2^ to 10^3^ MPa [17, 20, 38, 73–78] while stress in doubly clamped graphene ribbons or beams can reach 10^1^ MPa [7, 79–83] or about 200 MPa to 400 MPa in graphene ribbons with suspended silicon proof mass [72]. The built-in stress can substantially influence the resonance frequencies of resonators and accelerometers, as well as the force-induced deflection and strain in suspended 2D material membranes [72]. The fabrication process can further influence built-in stress, i.e., through design features, material growth, and the transfer material [73].

It should be noted that only a few of the materials listed in Table 1 have been shown to survive as self-suspended 2D material membrane, ribbon, or beam structure [3–5, 7]; however, many of these 2D materials may still be employed in NEMS sensors in form of multilayers or in combination with more stable suspended support layers such as graphene to form suspended heterostructures [63, 84, 85]. 2D materials may also be combined with polymer layers to form suspended membranes and beams [6, 86, 87]. The buckling metrology method has been recently revisited as an alternative method to determine Young's modulus of 2D materials and generally results in comparable experimental values as conventional metrology methods (where available) [88].

## 3. Fabrication Methods for Suspended 2D Material Devices

### 3.1. 2D Material Exfoliation and Growth

Initially, manual exfoliation of flakes from bulk crystals was the most popular fabrication method in 2D material research because it results in single crystalline nanosheets with low defect density. Although the method enables the fundamental exploration of material properties and new device concepts, it is not a process that can be scaled up to high-volume production for mass market applications. An alternative method to obtain larger quantities of 2D material is liquid-phase exfoliation in common solvents [89]. In this production method, guest molecules or ionic species are intercalated between layers of bulk crystals, increasing the interlayer spacing and reducing binding, thus facilitating exfoliation of monolayers in subsequent processes, such as ultrasonication [90], thermal shock [91], or shear [92]. Liquid exfoliation leads to dispersions of flakes that can be printed or sprayed onto substrates for sensor applications. This approach is suitable for example in applications, where the device functionality is mediated by mechanisms beyond the intrinsic material related to interfaces between the (randomly) oriented flake arrangement, i.e., binding flake edges in gas and chemical sensors or current percolation between flakes in piezoresistive strain sensing [93, 94].

In general, large-area chemical vapor deposited (CVD) graphene-related materials are the preferred option for integrated NEMS sensors, because the method is in principle compatible with semiconductor technology [13, 14] and has the potential to result in uniform, reproducible layers. CVD graphene is typically deposited on a catalytic surface such as Cu or Ni, from which it can be transferred to arbitrary target substrates and the number of layers is precisely controllable [95–99]. Wirtz et al. managed to fabricate gas tight large area membranes (4 cm × 4 cm) by stacking 3 or more CVD grown graphene layers [85]. The properties of CVD graphene strongly depend on the material quality, the substrate material on which the graphene sheet is placed, and the crystal grain size, which typically is on the order of a few *μ*m. Templated growth can lead to relatively large areas of crystalline CVD growth on copper [100] or sapphire wafers [101], although full wafer scale of singly crystal growth has yet to be demonstrated. Despite the grain boundaries, CVD graphene is not always inferior to exfoliated “perfect” graphene, depending on the application case [44, 102]. Other available forms of graphene include epitaxial graphene grown on SiC substrates. CVD is also widely used to grow other 2D materials on a large scale. A variety of different growth substrates are used depending on the targeted 2D material, for example, Si/SiO_2_, quartz, graphite, or even other 2D material substrates for the growth of MoS_2_, WS_2_, or WSe_2_ or metals such as copper, iron, or platinum for the growth of h-BN [85, 103–106]. However, the field of large area synthesis of 2D materials is until evolving rapidly. For example, it is challenging to obtain continuous films and to control the thickness and quality is far from mature. An extensive overview of the production and process challenges has recently been presented in Backes et al. [15].

An alternative synthesis approach introduced recently for transition metal dichalcogenides (TMDs) is thermally assisted conversion (TAC) utilizing vaporized chalcogenide precursors. For instance, Mo or more commonly MoO_3_ can be converted to MoS_2_ at high temperature [107–112]. This facile growth method is applicable to a wide range of TMDs, such as MoSe_2_ [113, 114], WS_2_ [115–117], WSe_2_ [118], PtSe_2_ [119], or PtTe_2_ [120]. The method yields continuous polycrystalline films, and therefore, prepatterned transition metals can be directly converted to structured TMDs. The thickness of converted TMDs is determined by the thickness of initial transition metal layers. Thus, the TAC synthesis has advantages in terms of manufacturability of NEMS sensor devices.

### 3.2. Fabrication of Devices with Suspended Membranes

**Figure 1:**
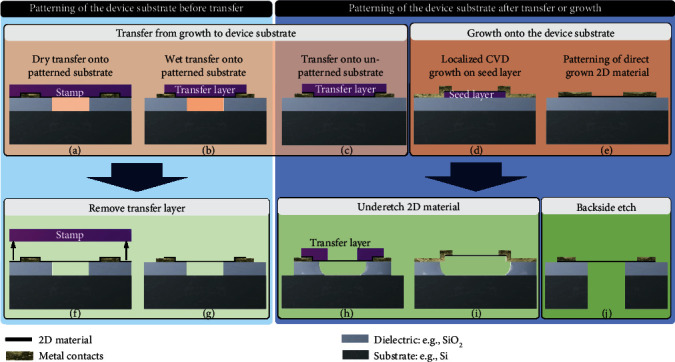
2D NEMS device fabrication methods. (a–e) Create a 2D material layer on the device substrate, where for (a) and (b) the device substrate is prepatterned and for (c–e) the substrate is patterned afterwards. (f)–(j) show post 2D material layer fabrication steps to create suspended membranes.

**Figure 2:**
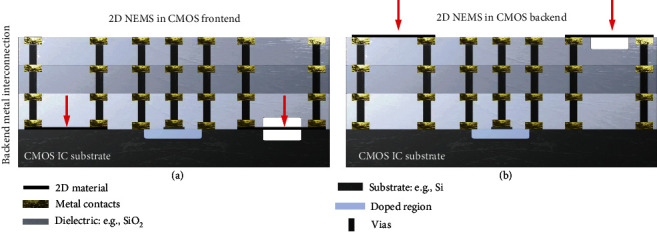
CMOS integration of 2D NEMS sensors in backend. (a) NEMS sensor devices integrated in the backend with interconnect layers stacked on top and frontend. (b) Integration of the 2D material in the frontend on top of the interconnect layers. The silicon IC substrate (dark grey) with transistors (blue) and interconnect metals (gray/yellow) is shown. Red arrows indicate the location of the black suspended graphene.

There are several routes to fabricate devices with suspended membranes (often called “drums”), beams, or ribbons of 2D materials. These routes can be distinguished by (1) the method of 2D material application (2D material transfer from the growth substrate to a target substrate in contrast to 2D material growth directly on the target substrate as shown in red color in Figures 1(a)–1(e)) and (2) the method of creation of cavities below the membranes (etching underneath the 2D material in contrast to 2D material transfer onto a preetched cavity, as shown in green color in Figures 1(f)–1(j)).

Figures 1(a) and 1(b) show the option where the device substrates are fabricated before 2D material transfer. This includes the etching of cavities over which the 2D material is to be suspended, as well as the fabrication of electrical contacts, gate electrodes, or sensing electrodes. Subsequently, 2D materials are transferred and suspended using wet transfer [121] or dry transfer using PDMS stamps [122], frame-based [99, 122–125], or other methods [126], each with its advantages and disadvantages [84]. It should be noted that compared to conventional transfer, transfer of 2D materials over cavities is challenging. Stamp transfer (Figure 1(f)) can fail by delamination due to low adhesion forces, rupture of the membranes at cavity edges, and stiction on the cavity bottom [127]. Alternatively, the transfer layer can be removed by etching (Figure 1(g)), which poses other challenges. The application of pressure on the stamp can affect the value and uniformity of the pretension in the suspended membrane and thus influence its mechanical resonance frequency and stiffness. Moreover, nonuniformity of the strain in the transfer layer can lead to wrinkled graphene membranes, and polymeric residues of a few nanometers from the stamp can be present [128]. In general, few-layer membranes are more stable, show a higher yield of intact membranes after fabrication [127], and can be suspended across larger areas.

After the 2D material is successfully suspended using dry (Figures 1(a) and 1(f)) or wet (Figures 1(b) and 1(g)) transfer, it is important to minimize the impact of subsequent process steps in order to reduce the risk of damaging the membrane and decreasing the yield of suspended 2D material membranes [84]. Process steps involving liquids suffer from capillary effects during drying and evaporation of the liquids, which typically decreases the yield of intact membranes [84]. Critical point drying (CPD) helps in this respect, but cannot be applied to membranes that seal holes because the high CPD pressures of more than 50 bar outside pressure can break the membranes. Here, a “transfer last” method (Figures 1(a) and 1(f)) is an option to create sealed membranes as required for absolute or sealed gauge pressure sensors [129]. Another option is to seal the membrane at a later stage in the process [21]. Ribbons can be either structured on the growth substrate and then transferred with alignment routines [130] or have to be structured after suspension, which is technologically extremely challenging.

Some of the issues can be avoided by either growing [131, 132] or transferring unsuspended 2D materials directly on the device substrate [72, 133] (Figures 1(c)–1(e)). It can then be patterned and subsequently the membrane can be released by isotropically underetching (Figures 1(h) and 1(i)), by using a sacrificial layer [134–137] or by releasing the membranes from the backside (Figure 1(j)). The remaining through-hole can be left open or resealed after release [133, 138]. Process steps that avoid capillary forces during drying, such as CPD or hydrofluoric acid (HF) vapor etch, can be used to avoid stiction and increase the yield of intact suspended membranes. Cleaning procedures for suspended 2D material devices are very delicate, because traditional methods used in MEMS manufacturing, such as ultrasonic-assisted dissolving or oxygen plasma ashing, are aggressive towards suspended 2D materials, and thus, these approaches are not suitable [137].

### 3.3. CMOS Integration

Eventually, it will become of interest to monolithically integrate suspended 2D materials with CMOS integrated circuits (ICs). Depending on the type of sensor and fabrication flow, the sensor can be integrated both in the front end (Figure 2(a)) and in the back end (Figure 2(b)) of the CMOS process. In both cases, devices with suspended 2D material membranes should be fabricated in a CMOS compatible way by growing the materials on wafer-sized substrates or by selective growth. The best process candidates are CVD and TAC, where the 2D material size is limited only by the reactor size. Wafer-scale transfer of graphene has been demonstrated and can in principle be integrated as a back-end-of-the-line process [139–143]. Direct growth of 2D materials in the back-end-of-the-line (Figure 2(b)) is only permitted if the growth temperature is below 450°C, which is for example possible for PtSe_2_ with a growth temperature of 400°C or less [119, 144]. To realize CMOS integration, many challenges still need to be addressed. In particular, front-end-of-the-line integration (Figure 2(a)) of suspended 2D materials is still very challenging [13], because the material needs to survive all subsequent CMOS process steps. Besides realizing high-yield methods for the process steps discussed above, compatibility to CMOS temperature budgets, material interactions, delamination requirements, low contact resistances, packaging methods, and reliability requirements will need to be dealt with.

Metrology is a general and ongoing challenge towards commercialization of 2D materials. This is augmented in membrane-based structures; scanning electron microscopy (SEM) is an option, but typically alters membrane properties due to the electron beam-assisted deposition of hydrocarbon molecules. Raman spectroscopy is a noninvasive method if applied with low laser power and can be extended to Raman tomography [145], which allows taking three-dimensional images of the entire device. Laser scanning microscopy is also feasible and noninvasive and can provide information about membrane deflection [146]. In addition, atomic force microscopy (AFM) [147], resonant interferometry [148], and colorimetry [149] can give useful information on the mechanical shape and stiffness of suspended 2D membranes.

## 4. Readout and Transduction Mechanisms

A number of electrical transduction mechanisms can be utilized for readout of 2D material NEMS sensors. Although optical readout and analysis techniques [7, 148] are very convenient and useful for fundamental studies, we focus here on electrical readout techniques since they are more easily and seamlessly integrated for practical NEMS sensor devices.

The main electromechanical transduction and readout techniques suitable for 2D material NEMS sensors are piezoresistive readout, capacitive readout, and transconductance readout. In addition, the electrical resistance of 2D material membranes can be used to sense changes in temperature, strain, carrier concentration, or mobility that are induced by surface interactions (e.g., gas adhesion causes doping of the 2D material). It is important to note that the electrical resistance of 2D materials, especially graphene, is extremely sensitive to various environmental parameters, which means that parameters such as small changes in the air humidity [150–153], light [154, 155], gases [119, 151, 152, 156], or temperature can strongly affect the electronic properties of a 2D material. Thus, for reliable use as sensors, these cross-sensitivity effects either have to be eliminated, by shielding or packaging, or they should be corrected for based on a calibration curve that eliminates environmental changes using input from a temperature or humidity sensor or reference device that is integrated in the same system [6, 25]. For resistance and Hall voltage measurements of 2D material NEMS sensors, it is important to realize low contact resistances and use high-mobility graphene, a general topic that receives considerable attention [157–162]. In the following, we now discuss the main electrical readout mechanisms of 2D sensors, piezoresistive, capacitive, and transconductance readout.

### 4.1. Piezoresistive Readout

The piezoresistive effect is defined as the change in electrical resistivity due to applied mechanical strain, which is related to the deflection of a membrane. The gauge factor (GF) is a measure for the piezoresistive effect [163]:

(1) $$\begin{array}{c} \text{GF}=\frac{\Delta R/R}{\Delta L/L}=\frac{\Delta R/R}{\varepsilon }=1+2\nu +\frac{\Delta \rho /\rho }{\varepsilon }. \end{array}$$

It is defined as the ratio of the change in the electrical resistance Δ*R* to the change Δ*ε* = Δ*L*/*L* in mechanical strain (change in absolute length). The geometric deformation is described by the term 1 + 2*ν*, with *ν* as Poisson's ratio. The gauge factor is directly related to the sensitivity of a piezoresistive sensor. Metals, such as constantan, which is used for commercial metal strain gauges, show a relatively low positive gauge factor of 2 [164]. Semiconductors, such as Si, have a gauge factor of -100 to 200 [165]. 2D materials show piezoresistive properties as well. Graphene has a gauge factor between 2 and 6 [25, 33, 34, 166], PtSe_2_ up to -85 [6, 144], and MoS_2_of -148, -224 and -40 for one, two and three layers [31, 167]. Simulations indicate a high gauge factor of up to 3000 for single-layer WSe_2_ [58] and around 1700 for single-layer MoSe_2_ [58]. These high values make piezoresistive readout an attractive method for readout of NEMS based on 2D materials. Moreover, piezoresistive readout can be scaled down well [168]. Interestingly, for resonant strain gauges with nanoscale dimensions, such as doubly clamped carbon nanotubes, silicon nanowires, and graphene ribbons, the gauge factor of a strain gauge can be significantly amplified as a result of an asymmetric beam shape at rest [72, 169].

### 4.2. Capacitive Readout

Capacitive readout is an alternative method to determine the deflection of 2D membranes. For a deflection *δ*, the capacitance of a drum with area *A* and gap *g* is given by *C*_drum_ = *Aε*_0_/(*g* − *δ*). The responsivity therefore scales as *dC*/*dδ* = *Aε*_0_/*g*^2^ and increases by reducing the gap *g*. With respect to other deflection readout mechanisms, the important advantage of capacitive readout is that the capacitance only depends on the geometry of the structure, regardless of the membrane resistance and temperature. In practice however, it is difficult to fabricate membranes with gaps smaller than 100 nm with sufficient yield [127] without causing stiction during fabrication. Also, a small gap limits the maximum membrane deflection and thus the maximum dynamic pressure range of the device. An alternative approach to increase responsivity is therefore to increase the area of the membranes, for instance, by placing many graphene sensors in parallel [87]. Another challenge is that there are usually parasitic parallel capacitances *C*_par_ present between the top and bottom electrodes that need to be minimized to reduce power consumption and increase signal-to-noise ratio. This can be achieved by utilization of an insulating layer with a low dielectric constant and sufficient breakdown strength, a small overlap area between top and bottom electrodes (using local gates), and the utilization of an insulating, low dielectric constant substrate [87]. A unique feature of monolayer membranes, such as monolayer graphene with low carrier densities, is that their capacitance is lowered by an effective series quantum capacitance [170], especially close to the Dirac point. When a readout voltage *V*_g_ is applied across the sensor to determine its capacitance, this will not only affect the quantum capacitance but can also result in an electrostatic pressure *P*_el_ = *ε*_0_*V*_g_^2^/(*g* − *δ*)^2^ that adds to the gas pressure and deflects the membrane. These effects need to be considered to accurately operate capacitive graphene pressure sensors, either by proper modeling or by proper calibration.

### 4.3. Transconductance Readout

Transconductance readout is a sensitive electrical readout method for 2D material membranes (see, e.g., [171, 172]). It requires a three-terminal geometry, in which the conductivity of the 2D membrane is measured between a source and drain electrode, while a voltage is placed on a nearby gate electrode. When the membrane is deflected, the capacitance between gate and membrane changes and results in a different charge *Q* on the membrane (*Q* = *CV*_g_), which results in a change in charge density and thus a different conductivity of the membrane, similar to that in the channel of a field-effect transistor.

### 4.4. Readout of Resonant Sensors

For resonant sensors, usually a vector network analyzer or spectrometer is used to determine the resonance frequency from a frequency spectrum or the transfer characteristic. In order to continuously monitor a resonance frequency, the resonant sensor can be configured in a direct feedback loop as a self-sustained oscillator that generates a signal with a sensor signal-dependent frequency that can, for example, simply be read out by a digital frequency counter circuit that counts the number of zero-crossings per second. This method has been applied successfully to MEMS squeeze-film pressure sensors [173]. In more advanced implementations, readout can be performed using phased locked loops [174]. Nevertheless, the feasibility of realizing an integrated portable resonant graphene sensor still needs to be proven.

### 4.5. Actuation Methods

Actuation methods for 2D membranes include electrostatic actuation, opto- or electrothermal actuation [21, 175–178], hydraulic pumping [179], mechanical amplification [180], and piezoelectric excitation [180, 181]. In general, for realizing most types of sensors concepts, the challenge is more in the readout than in the actuation. Nevertheless, for sensors that utilize actuation voltages and currents, these need to be stable and noise-free, since any drift and noise at the actuation side will end up in the readout signal. The effects of noise can be mitigated by using a longer time-averaging or by placing membranes in parallel to increase responsivity [87, 182].

## 5. Mechanical Properties of Suspended 2D Material Membranes and Ribbons

2D material membranes and ribbons, specifically those made from graphene, can be made a factor 1000 thinner than those of current commercial MEMS sensor membranes or beams. As a consequence, these graphene membranes and ribbons have a much lower flexural rigidity. This allows either the reduction of the sensor size to only a few microns in diameter or side length while retaining the flexural softness of the membrane or beam or a significant increase in sensor responsivity. However, to enable these, several challenges need to be tackled. The membrane/ribbon deflection needs to be determined with nanometer precision using accurate transduction mechanisms and the pretension *n*_0_ in the graphene needs to be low enough to ensure that the responsivity is not limited by it. For the deflection of a doubly clamped 2D material ribbon caused by a center point force, the deflection at the center of the ribbon is described by

(2) $$\begin{array}{c} F=16\left(\frac{EWH^{3}}{L^{3}}\right)Z+8\left(\frac{EWH}{L^{3}}\right)Z^{3}+4\left(\frac{T}{L}\right)Z, \end{array}$$

where *F* is the load applied at the center of the ribbon, *Z* the resulting deflection of the ribbon at its center (for large deflection with respect to the thickness of the ribbon), *E* the Young's modulus of the 2D material, *W* the width of the ribbon, *H* the thickness of the ribbon, *L* the total length of the ribbon, and *T* the built-in tension force of the ribbon [72]. Another aspect of 2D material membranes and ribbons that is intrinsically different from conventional devices is that the force-deflection curve of indentation experiments tends to become nonlinear at much smaller deflections than for bulk materials, due to the small thickness and high Young's modulus in graphene in combination with geometric nonlinearities (from the second term on the right-hand side of equation (2)) related to membrane stretching. This effect increases the stiffness and reduces the sensor linearity, which in principle can be corrected by proper calibration. It will increase operation range but reduces responsivity and will therefore require tradeoffs between dynamic range and responsivity [182]. Since graphene membranes and ribbons have a much smaller area, they feature higher thermomechanical “Brownian motion” noise [177] that translates, for example, for a circular membrane to a pressure noise *p*_*n*_:

(3) $$\begin{array}{c} p_{n}^{2}=\frac{4k_{B}T\omega_{0}m_{\text{eff}}}{A^{2}Q}\left[\frac{{Pa}^{2}}{Hz}\right], \end{array}$$

where *T* is the temperature, *Q* the quality factor, and *ω*_0_ the resonance frequency of the membrane. This equation shows that on the one hand 2D material pressure sensors have reduced noise due to their small effective mass *m*_eff_, whereas on the other hand thermomechanical noise will increase as a consequence of their smaller area and higher resonance frequency. Nevertheless, it is often not the thermomechanical noise that limits NEMS sensor resolution in practice, but readout noise.

A further requirement on membrane properties in many NEMS sensors, such as in some pressure sensor, is that the membrane may need to be hermetically sealed, such that the pressure in the reference cavity is constant and gas leakage is negligible during its lifetime [21]. Despite the impermeability of graphene for gases [20, 22], it was found that gas can leak via the interface between the substrate and the graphene. This leakage path needs to be sealed for long-term pressure stability inside the reference cavity [21]. In pressure sensing applications, it is typically preferred to maintain a vacuum or a very low gas pressure environment in the cavity below the 2D material membrane, to avoid internal pressure variations with temperature according to the ideal gas law, or alternatively, methods to correct for these using an integrated temperature sensor are required.

## 6. 2D Material NEMS Sensors

### 6.1. Pressure Sensors

Silicon-based pressure sensors were the first microelectromechanical system (MEMS) product to reach volume production [183]. The number of pressure sensors produced per year currently exceeds a billion units per year. Whereas the field of pressure sensing also includes liquid, tactile, and touch sensing applications, we focus here on gas pressure sensors using suspended membranes, with main applications as altimeters, barometers, gas control, and indoor navigation. MEMS pressure sensors usually determine the pressure from the pressure difference Δ*p* (see equation (1)) across a plate that induces a deflection *δ* = *α*Δ*pA*^2^/*t*^3^, a geometry and material dependent factor *α*.

Commercial MEMS sensors can resolve pressure differences as small as 1 Pa, corresponding to altitude changes of only 5 cm. To reach this resolution, an extremely low stiffness of the mechanical plate is required, resulting in diaphragm sizes of several hundreds of microns at membrane thicknesses in the order of 0.5-10 *μ*m. In addition, highly sensitive membrane deflection detection circuitry is used, conventionally based on piezoresistive readout, but recently also capacitive readout, such as the SBC10 pressure sensor of Murata with a responsivity of 55 fF/kPa [184]. Reducing the size and improving the sensitivity of pressure sensors are generally of interest. For example, size may be a decisive form factor for wearable electronics. Enhanced sensitivity of 2D sensors may also enable new applications that are currently not feasible, like altimeters with sub-cm resolution for indoor navigation or pressure sensors for presence detection. Moreover, higher sensor sensitivity can reduce size, acquisition time, power consumption, and cost of readout electronics.

In the following, we will first discuss two types of static graphene pressure sensors: piezoresistive and capacitive pressure sensors. Then, we will discuss two types of resonant pressure sensors and Pirani pressure sensors. Finally, we will compare the different types of pressure sensors.

#### 6.1.1. Piezoresistive Pressure Sensors

**Figure 3:**
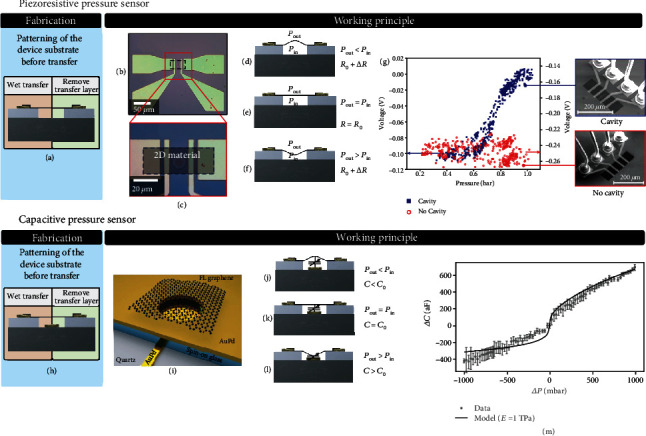
Piezoresistive NEMS pressure sensor. (a) Fabrication method of the suspended membrane (according to Figure 1). (b, c) Example device image [6]. (d–f) Working principle: pressure difference causes tension which alters the membrane resistance by the piezoresistive effect. (g) Graphene piezoresistive pressure sensor measurement [25]. Capacitive pressure sensor: (h) fabrication of the suspended membrane. (i) Device schematic [182]. (j–l) Working principle: a pressure difference causes the membrane to deflect and alter the capacitance between the graphene and the bottom electrode. (m) Device measurement [182].

The basic geometries and the operation principles of 2D piezoresistive pressure sensors are shown in Figures 3(a)–3(c) and Figures 3(d)–3(f), respectively. The first subfigures (Figures 3(a) and 3(h)) show the device fabrication according to methods described in Figure 1 (coloring shows 2D material transfer or growth and method to suspend the membranes). When the membrane is bent by a pressure difference, it introduces strain into the material (Figures 3(d)–3(f)) which is detected as a resistance change (Figure 3(g)). It is important to note that gasses or moisture that is in contact with the suspended 2D material membrane typically affects its resistance, which can interfere with the piezoresistive signal during pressure measurements [25, 35, 151]. In addition to self-suspended graphene membranes [25], graphene resistors have been used to piezoresistively detect the motion of membranes made from SiN [185] or polymers [186]. Even though graphene enables very thin membranes, its piezoresistive gauge factor GF = (Δ*R*/*R*)/*ε* is relatively low (see Table 1) [25, 35]. Other 2D materials have higher gauge factors (see Table 1) and are promising for improving piezoresistive pressure sensor sensitivity, as demonstrated for PtSe_2_ [6]. The membrane area of graphene [25] and PtSe_2_ [6] devices can be reduced to around 170 *μ*m^2^, which is significantly smaller than the area (90000 *μ*m^2^) of conventional MEMS pressure sensors [172, 187]. Low-dimensional materials, such as carbon nanotubes [188, 189] or silicon nanowires [10, 190], can also be used for piezoresistive sensors, due to their high GFs [191]. However, these materials can only be used as sensing elements and usually need a separate membrane to support them, in contrast to 2D membranes that can have both a mechanical and electrical function. Such purely 2D material membranes combine a very thin membrane with the intrinsic readout mechanism and potentially enable up to four orders of magnitude smaller device footprints [6, 25].

#### 6.1.2. Capacitive Pressure Sensors

2D capacitive pressure sensors (Figures 3(h) and 3(i)) consist of a capacitor, which is formed between the membrane and a bottom electrode, such that a pressure change results in a capacitance change (Figures 3(j)–3(l)). As can be seen in Figure 3(m), the capacitance is a nonlinear function of pressure. This is both due to the nonlinearity in the capacitance-deflection relation and due to the nonlinearity in the pressure-deflection curve (equation (3)). Main parameters that can influence the shape of this curve are the gap size, membrane thickness, Young's modulus, pretension, membrane radius, and quantum capacitance. As can be seen from the slope of the curve in Figure 3(m), the sensor is most sensitive when the pressure difference across it is zero.

When a capacitive pressure sensor is made out of a single graphene drum, its capacitance and change in capacitance is very small. For readout, it requires detecting a small capacitance change on a large parasitic background capacitance. Even when using insulating quartz substrates to reduce the parasitic capacitance [182], it is difficult to measure the capacitance changes, since responsivities of a drum with a 5 micron diameter are at most 0.1 aF/Pa, which at a voltage of 1.6 V corresponds to only 1 electron moving onto the graphene for a pressure change of 1 Pa. By utilizing a high-frequency AC signal to charge and discharge the capacitor many cycles, signal-to-noise ratios can be improved to achieve a resolution of 2-4 aF/√Hz, requiring at least 20-40 of these drums in parallel to reach a pressure resolution of 1 Pa with an acquisition time of 1 second [192]. Recently, capacitive pressure sensors have been reported with many graphene drums in parallel that outperform the best commercial capacitive pressure sensors (SBC10 of Murata, responsivity 55 aF/Pa [184]) and that could be read out using a commercial IC [193]. With a large 5-layer graphene membrane, a responsivity of 15 aF/Pa was reached [194] and an even higher responsivity of 123 aF/Pa was reached with graphene-polymer membranes [87]. Increasing drum diameter or further gap or tension reduction can also improve responsivity of graphene pressure sensors, although these options come with significant engineering challenges.

#### 6.1.3. Tension-Induced Resonant Pressure Sensors

**Figure 4:**
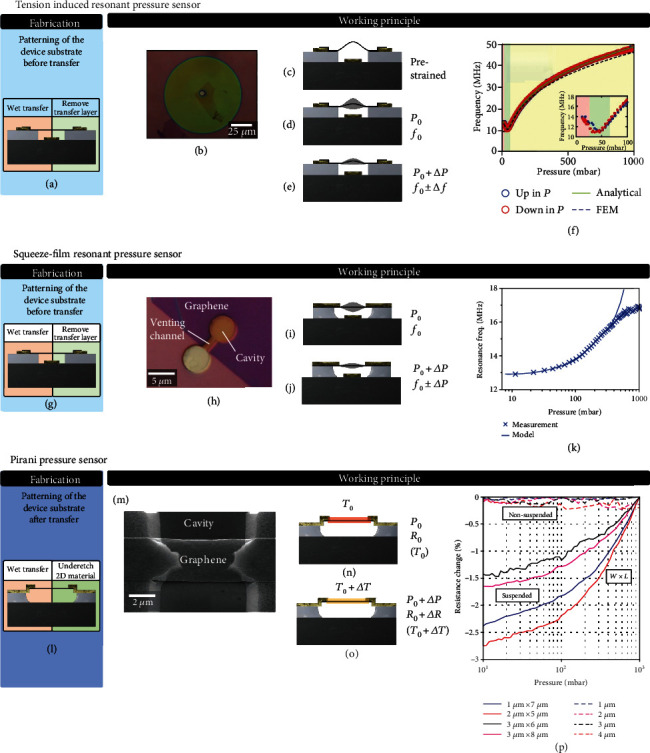
Tension-induced pressure sensor: (a) fabrication method of the suspended membrane (according to Figure 1) and (b) example device [21]. (c–e) Working principle: the gas pressure difference across the membrane causes a membrane deflection and tension change that is measured via the resonance frequency. (f) Graphene tension-induced pressure sensor measurement [21]. Squeeze-film pressure sensor: (g) fabrication of the suspended membrane and (h) example device [175]. (i, j) Working principle: the stiffness and compressibility of the gas under the membrane increases the stiffness of the membrane that is measured via the mechanical resonance frequency. (k) Example measurement of a graphene-based squeeze-film pressure sensor [175]. Graphene Pirani pressure sensor: (l) fabrication of the suspended membrane; (m) example device of a Pirani pressure sensor [132]. (n, o) Working principle: the temperature, and temperature-dependent resistance, of the suspended, Joule-heated graphene beam depends on the pressure-dependent gas cooling rate. (p) Example measurement of a Pirani pressure sensor based on graphene [132].

Resonant tension-induced pressure sensors, similar to piezoresistive pressure sensors, monitor the effect of gas pressure on the strain in a membrane. However, here, the change in strain is monitored via its effect on the resonance frequency of the graphene membrane (Figures 4(a) and 4(b)). Bunch et al. [20] first utilized this effect to characterize the pressure difference across sealed graphene membranes in 2008. This demonstration of the extreme sensitivity of the resonance frequency to pressure was later confirmed with sealed graphene [21] and MoS_2_ [195] membranes, resulting in variations in the fundamental resonance frequency of more than a factor of 4 (Figures 4(c)–4(f)). A theoretical analysis of the dependence of the resonance frequency of a circular membrane on pressure found that the values of Young's modulus that were extracted from the experimental fits are anomalously low [21]. It is still unclear whether this is related to wrinkling effects [196], deviations from the theoretical shape and tension, or squeeze-film, slippage, or delamination effects. Also, the pressure dependence of the quality factor of tensioned membranes is not fully understood [136] and might not only depend on the pressure difference but also on the individual gas pressures below and above the membrane.

Typical responsivities *dω*_0_/*dp* are larger than 200 Hz/Pa. It typically takes 1/200 second to determine a frequency change of 200 Hz; therefore, this indicates that it might be possible to resolve pressure changes of 1 Pa in less than 5 ms. To actually achieve this, temperature [176], mass loading, and other effects that affect the resonance frequency of the membrane need to be prevented or corrected with proper calibration using additional sensors. The low *Q* (*Q* of approximately 3) of graphene at atmospheric pressure will increase the power and time required to accurately determine the resonance frequency.

It should be emphasized that the high responsivity of tension-induced pressure sensors can be attributed to the extreme thinness of graphene, which results in a low mass and thus in a very high initial resonance frequency *ω*_0_, but also in a relatively large strain and related tension-induced resonance frequency changes when the graphene “balloon” is inflated.

#### 6.1.4. Squeeze-Film Resonant Pressure Sensors

A second type of resonant pressure sensor is the squeeze-film pressure sensor. In contrast to the previously discussed sensors, squeeze-film pressure sensors do not require a hermetically sealed cavity (Figures 4(g) and 4(h)). The operation mechanism is based on the measurement of compressibility of gas inside the cavity under the graphene membrane. The compression occurs when the time it takes for pressure in the cavity to equilibrate is much longer than the period of the motion of the membrane, effectively trapping the gas in the cavity. It follows from the ideal gas law that the resonance frequency is *ω*_res_^2^ = *ω*_0_^2^(*P* = 0) + *A* *P*/(*m* *g*), where *m* is the membrane mass, so the low areal mass density of graphene is an advantage that increases the responsivity Δ*ω*_res_/Δ*P* of the sensor. The change in the resonance frequency with respect to the vacuum value *ω*_0_ is dependent on the mass and geometry of the graphene cavity (Figures 4(i) and 4(j)). It has been shown [175] that the small graphene thickness and cavity depth result in a frequency change as large as 10-90 Hz/Pa, which is a factor of 5-45 higher than that in conventional MEMS squeeze-film sensors despite the smaller area of the device (Figure 4(k)). More recently, the feasibility of fabricating squeeze-film pressure sensors using transferless graphene (Figure 1(d)) has been demonstrated [132].

#### 6.1.5. Pirani Pressure Sensors

Pirani pressure sensors operate by measuring the pressure-dependent thermal conductivity of the surrounding gas via its influence on the temperature-dependent resistance of a suspended membrane (Figures 4(l) and 4(m)). In contrast to all other pressure sensors discussed above, the Pirani sensor does not mechanically move during operation. Conventionally, Pirani sensors are only used in vacuum systems. However, in [197], it was shown that the sensitivity range of these sensors can be brought to atmospheric pressure by reducing the gap down to 400 nm. The advantage of using graphene for Pirani sensors is that it takes much less power to heat a thin beam than a thick beam, and the temperature of the graphene beam depends more strongly on the cooling by surrounding gases due to its large surface-to-volume ratio (Figures 4(n)–4(p)). With a transferless process flow (Figure 1(d)), the feasibility of graphene Pirani pressure sensors was recently demonstrated [132]. It should be noted that the response of Pirani pressure sensors is gas dependent, due to differences in thermal conductivity of different gases. This property might be employed to utilize the Pirani sensor as a gas sensor, when complemented by a pressure sensor that is independent of the type of gas.

#### 6.1.6. Pressure Sensor Comparison

Important benchmark parameters for comparing different pressure sensors include size, power consumption, acquisition time, cross-sensitivity, reliability, and production cost. In terms of performance, the capability to detect small pressure changes Δ*P* is an important parameter to compare the different sensors. To detect the signal of such a small change, it needs to be larger than the pressure noise in the system, i.e., the signal-to-noise-ratio (SNR) needs to exceed 1. Usually, the electrical readout noise (Johnson-Nyquist) is the dominant noise source that limits the SNR in these systems [198]. For a pressure change Δ*P*, the SNR is determined to compare the different types of pressure sensors (piezoresistive, capacitive, and squeeze-film). The noise in a capacitive pressure sensor can be determined by using the charge noise of the capacitor $\sigma_{Q}=\sqrt{4\;k_{B}TC}$ and the total energy costs for a measurement *E*_tot_ = *Pt*_readout_ = *NCV*^2^, where *k*_*B*_ is the Boltzmann constant, *T* the temperature, *C* the capacitance, *P* the electrical power consumption, *t*_readout_ the readout time over which the measurement results are averaged, *V* the voltage, and *N* the number of measurements [198]:

(4) $$\begin{array}{c} \text{Noise}=\sigma_{C}=\frac{\sqrt{4\;k_{B}TC/N}}{V}=C\;\sqrt{\frac{4k_{B}T}{Pt}}. \end{array}$$

The noise itself does not depend on the responsivity, but the capacitive signal *dC* = Δ*P* *dC*/*dP* does depend on the pressure change Δ*P* as well as the responsivity. By taking the ratio, the SNR can be calculated for the capacitive pressure sensor defined as

(5) $$\begin{array}{c} \text{SN}{\text{R}}_{\text{CAP}}=\frac{1}{C_{0}}\;\frac{dC}{dP}\sqrt{\frac{Pt_{\text{readout}}}{4k_{B}T}}\;∆P. \end{array}$$

Here, *C*_0_ is the capacitance in the unloaded state. Note that the minimum detectable pressure change corresponds to solving this equation for Δ*P* for SNR = 1. For comparison, the SNR can be determined for a piezoresistive pressure sensor. An expression like (5) is found, with the term 1/*C*_0_ × *dC*/*dP* being replaced by 1/*R*_0_ × *dR*/*dP* for piezoresistive pressure sensors [198]. In case of the squeeze-film pressure sensor, a factor *Q* needs to be added resulting in 1/*C*_0_ × *dC*/*dP* being replaced by 2/*ω*_0_ × *dω*_res_/*dP* × *Q*. We assume *Q* = 3 for graphene at atmospheric pressure [199].

**Figure 5:**
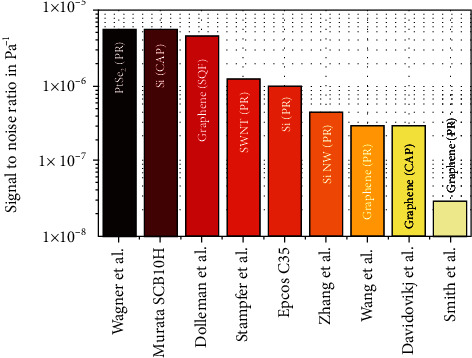
SNR comparison of piezoresistive (PR), capacitive (CAP), and squeeze-film (SQF) MEMS pressure sensors. Included are Wagner et al. [6], Murata SCB10H [184], Dolleman et al. [175], Stampfer et al. [188], Epcos C35 [200], Zhang et al. [172], Wang et al. [185], Davidovikj et al. [182], and Smith et al. [25].

With these rough estimates of the SNR, based on an optimal performance of the readout system, different pressure sensor types can be directly compared to each other, which are shown in Figure 5. An SNR of 5.5 × 10^−6^ Pa^−1^ was calculated for both the PtSe_2_ membrane-based piezoresistive by Wagner et al. [6] and the commercial capacitive pressure sensors Murata SCB10H [184], which shows one of the highest SNR values available. The graphene membrane-based squeeze-film by Dolleman et al. [175] and capacitive pressure sensor by Davidovikj et al. [182] show values of 4.7 × 10^−6^ Pa^−1^ and 0.3 × 10^−6^ Pa^−1^, respectively. A SNR of 0.3 × 10^−6^ Pa^−1^ and 0.3 × 10^−7^ Pa^−1^ could be calculated for the piezoresistive graphene-based sensor by Wang et al. [185] and by Smith et al. [25], respectively. These 2D material sensors were also compared to other low-dimensional material-based NEMS pressure sensors (carbon nanotubes, Stampfer et al. [188]; silicon nanowires, Zhang et al. [172]) as well as to another commercial sensor, Epcos C35 [200], which is summarized in Figure 5. The PtSe_2_ sensors show a factor of 5 to 200 higher SNR and up to 5 orders of magnitude smaller sensor area in comparison to state-of-the-art pressure sensors.

## 7. Graphene Microphones

A microphone is essentially a pressure sensor that operates at audible or ultrasound frequencies. Similar to pressure sensors, the extreme thinness and the resulting flexibility of suspended 2D materials make them highly susceptible to sound pressure variations and thus suitable for application as microphones. In the last decades, MEMS microphones have replaced most conventional microphones in mobile devices and have become a billion-dollar market, where often multiple microphones are employed for realizing directionality and noise cancellation. The key advantage of using suspended graphene as a microphone membrane is its low stiffness *k*_eff_. In conventional microphones, the stiffness cannot be lowered much further, because for a flatband frequency response it is required to have a resonance frequency *ω*_2_ = *k*_eff_/*m*_eff_ that exceeds the audible bandwidth (usually >20 kHz). Since graphene is extremely thin, it has a very small mass, allowing low stiffness to be combined with a high resonance frequency, offering interesting prospects for enabling wide bandwidth microphones that can detect small sound pressures. In addition, the low mass of graphene might be advantageous to reduce the pressure noise level based on equation (3). Besides improved performance, the advantages of graphene can also be utilized for area downscaling of microphones while maintaining current performance. This in turn can facilitate low-cost arrays of microphones that can enable directionality and might find applications in 3D ultrasound imaging and noise cancellation. Challenges in reaching sufficient signal-to-noise ratio are even much tougher in microphones than in pressure sensors since current typical MEMS microphones boast responsivities (sensitivities) of >10 mV/Pa and impressive pressure noise levels below *p*_*n*_ < 10 *μ*Pa/√Hz [201]. This low-noise, high-responsivity performance has not yet been demonstrated with graphene membranes, but theoretically, graphene is expected to outperform conventional MEMS membranes according to equation (3).

**Figure 6:**
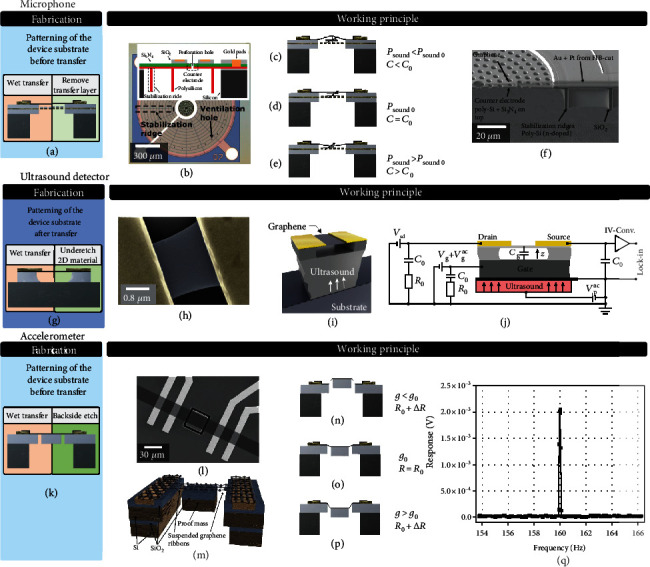
Microphone: (a) fabrication method of the suspended membrane (according to Figure 1); (b, f) images of an example device [146]. (c–e) Working principle: the sound pressure-dependent deflection of the membrane is detected via its capacitance with respect to the backplate. Ultrasound detector: (a) fabrication of the suspended membrane; (h) example device [181] and (i, j) working principle: the graphene membrane is moved by the ultrasound-induced motion of its supports, and its motion is detected using transconductance readout. Accelerometer: (k) fabrication of the suspended membrane; (l, m) example device [72], (n–p) working principle: the acceleration-induced forces on the suspended mass cause tension in the graphene that is detected using the piezoresistive effect. (q) The output signal of an accelerometer [72].

Condenser microphones with multilayer graphene membranes (20-100 nm thick) were reported with radii varying from 12 mm down to 40 *μ*m [146, 202, 203]. These devices cover a frequency range from the audible domain [202, 203] up to the ultrasonic domain [146]. Devices with a small membrane diameter (Figures 6(a)–6(f)) [146] operate over a wide frequency range that includes ultrasonic frequencies, while requiring low voltages, below the pull-in voltage of 1.78 V, which is well suited for use in mobile phones that provide a standard supply voltage of 2 V. Devices with a large membrane diameter [202, 203] require higher operation voltages but were also shown to function as a speaker. Importantly, some of the reported devices outperform high-end commercial nickel-based microphones over a significant part of the audio spectrum, with a larger than 10 dB enhancement of sensitivity, demonstrating the potential of graphene in microphone applications. Compared to conventional MEMS microphones with sensitivities of approximately -36 dB (around 15.8 mV/Pa), a supply voltage of 1.62-3.6 V [204], and an active membrane of 5 mm^2^ [205], graphene-supported microphone diaphragms have sensitivities of up to 10 mV/Pa, at a supply voltage of 1 V, and a diaphragm size of 38.22 mm^3^ [206]. Thus, current silicon-based microphone technologies are even more sensitive than those using graphene, but microphone designs with two vibrating membranes are usually used to amplify the signal [205], which is currently not the case with graphene.

## 8. Ultrasound Detection

Recently, graphene-based high-frequency geophones have been introduced to detect ultrasonic waves in a silicon substrate [181] and to detect generalized Love waves in a polymer film (Figures 6(g)–6(j)) [207]. In these works, a highly sensitive electronic readout was employed reaching a resolution in ultrasonic vibration amplitude of 7 pm/√Hz. Interestingly, this resolution is independent of the mechanical resonance frequency of the suspended graphene membrane. The coupling mechanism between the substrate vibrations into the graphene membrane is currently still under debate, as the detected amplitudes are seemingly large. Recent work using an interferometric detection scheme suggests that graphene not just acts as a detector of the ultrasonic vibrations and resonant modes in the substrate but also as an amplifier [180]. However, the physical origin of the strong coupling remains elusive. The possibility of using graphene for detecting vibrations or sound in solids could enable a new regime of ultrasound imaging at higher frequencies and smaller wavelengths than currently possible.

## 9. Accelerometers

In current silicon-based MEMS accelerometers, the springs and interdigitated readout electrodes cause a significant increase in the device area. On the one hand, this is caused by the requirement of a sufficiently small spring constant, which requires long compliant springs. On the other hand, for capacitive readout MEMS accelerometers, a sufficient capacitor area is required, which results in many interdigitated readout electrodes. Graphene and 2D materials on their own are not well suited for accelerometers, because their intrinsic mass is too small to achieve sufficient responsivity. 2D materials thus require an additional proof mass in the suspended region, which is displaced by acceleration forces. Although graphene has a small piezoresistive gauge factor, it can exhibit a large resistance change per Newton force (1/*F* × Δ*R*/*R*), because of its ultimate thinness. Its high Young's modulus and fracture strain further suggest that it is suitable for suspended devices with attached proof masses. Figures 6(n)–6(p) show an example of such a graphene NEMS accelerometer design, where the graphene simultaneously forms the springs of the spring-mass system and the piezoresistive transducer elements. The strain in the suspended graphene ribbons or membranes resulting from acceleration causes resistance changes in the graphene, due to the piezoresistive readout technique used in the accelerometers.

Double-layer graphene ribbons with large suspended silicon proof masses were realized with a conventional MEMS and NEMS manufacturing approach [72]. The graphene was suspended by dry etching followed by vapor HF etching to remove a sacrificial buried oxide layer (similar to Figure 1(h)). The suspended silicon proof masses had dimensions of up to 50 *μ*m × 50 *μ*m × 16.4 *μ*m (Figures 6(k)–6(m)), which is more than three orders of magnitude heavier than the masses deposited on previous devices [208–210]. The graphene ribbons with suspended proof mass occupy at least two orders of magnitude smaller die areas than conventional state-of-the-art silicon accelerometers while keeping competitive sensitivity (Figures 6(n)–6(q)) [72]. After normalization, the relative responsivity (resistance change per proof mass volume) in graphene ribbon accelerometers is at least one order of magnitude larger than the silicon state of the art. This demonstrates the potential to shrink the size of graphene-based NEMS accelerometers and gyroscopes despite graphene's low gauge factor.

The sensitivity of graphene accelerometers can be further improved by increasing the attached mass or by reducing the width of the suspended graphene [72]. From the perspective of material selection, the use of other two-dimensional materials like MoS_2_ [29, 31, 36] or PtSe_2_ [6, 144] with significantly higher piezoresistive gauge factors would also potentially improve the device sensitivity, although these materials need to be carefully evaluated with respect to their mechanical stability and adhesion force to the substrate. To this end, device designs based on fully clamped membranes improve the mechanical robustness by avoiding edges that are starting points for tearing under stress. However, this approach is a compromise as the signal response of fully clamped membranes is generally lower than that of ribbons with identical proof masses and trench width due to the lower strain levels and parasitic parallel resistances [133].

In addition to the above-mentioned demonstrations of graphene NEMS accelerometers, there are a limited number of experimental realizations of suspended graphene membranes or ribbons with attached proof masses. Micrometer-sized few-layer graphene cantilevers with diamond allotrope carbon weights fabricated by focused ion beam deposition have been used to study the mechanical properties of graphene [208]. A kirigami pyramid was combined with cantilevers made of suspended graphene and supported 50 nm thick gold masses, but these devices had to be kept in liquid to maintain their mechanical integrity [209]. Finally, suspended graphene membranes circularly clamped by SU-8 that are supporting a mass made of either SU-8 or gold located at the center of the graphene membranes and that were evaluated as shock detector for ultrahigh mechanical impacts [210]. These reports utilized very small masses and some employed fabrication methods that are not considered compatible with semiconductor manufacturing. In addition, graphene-based resonant accelerometers have been proposed on theoretical grounds but not yet experimentally demonstrated [211–213]. In these concepts, the acceleration would act on suspended graphene beams or membranes, thereby resulting in added strain in the suspended graphene beams or membranes, thus causing a related shift in their resonance frequencies.

## 10. Hall Sensors

**Figure 7:**
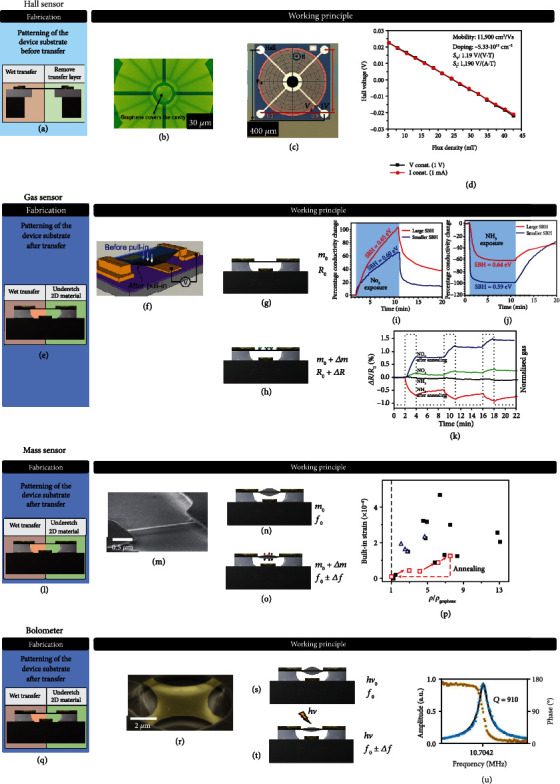
Hall sensor: (a) fabrication method of the suspended membrane (according to Figure 1) and (b, c) example device [146] and readout of an example device [146]. Gas sensor: (e) fabrication of the suspended membrane and ribbon and (f) example device [244]. (g, h) Working principle: gas molecules adhere to the (functionalized) 2D material and alter its resistance via electronic or chemical interactions. (i, j) Readout of an example device [248] and (k) typical sensor response plot of MoSe_2_ sensors depending on electron-donating/withdrawing gas [110]. Mass sensor: (l) fabrication of the suspended membrane, (m) example device, and (n, o) working principle: by measuring the resonance frequency, the mass change of the membrane is derived. (p) Extracted mass and tension of the membrane during multiple loading cycles [83]. Bolometer: (q) fabrication of the suspended membrane and ribbon and (r) example device [266]. (s, t) Working principle: when radiation heats the membrane, this alters its tension and causes a shift in mechanical resonance frequency. (u) Readout of an example device with a graphene membrane [266].

When a conductor that is biased on one side is exposed to an external magnetic field, charge carriers experience a Lorentz force that drives them in a direction perpendicular to the electric field and the external magnetic field. The resulting Hall voltage is a measure of the magnetic field and is proportional to 1/*n*, where *n* is the charge carrier concentration. The electronic structure of single-layer graphene results in a very low carrier density at the minimum of its conductivity and thus high Hall voltage. In addition, the charge carrier concentration can be tuned to reach high responsivity. The ultimate signal-to-noise ratio of Hall sensors is proportional to the mobility *μ*. The very low effective mass of charge carriers in graphene translates into very high mobility at room temperature, which enables high-performance graphene-based magnetic field sensors. The mobility in graphene depends to a large extent on the (dielectric) environment, i.e., the interface with its surroundings. Relevant to this review, high mobilities of up to *μ* = 200000 cm^2^/Vs have been measured in suspended graphene [214–216], which are significantly higher compared to up to *μ* = 20000 cm^2^/Vs for supported graphene on a SiO_2_ substrate [217]. Suspended graphene Hall sensors are of interest (Figures 7(a)–7(d)) because the voltage sensitivity (SV) of linear Hall sensors depends on the charge carrier mobility *μ* (SV ∝ *μ*∙(*W*/*L*)), where *W* and *L* are the width and length of the device [218]. The carrier mobility of electrons is about 1241 cm^2^/Vs in silicon at a dopant concentration of approximately 1017 cm^−3^ at room temperature [219]. The intrinsic SV is thus approximately 160 times greater for suspended graphene (at *μ* = 200000 cm^2^/Vs) than for silicon. Also, graphene shows a linear Hall response over several hundred mT [220] and surpasses commercial Hall sensors based on silicon technology [221]. Nevertheless, commercial monolithic silicon Hall sensors produced with BiCMOS technology, such as the Infineon linear Hall sensor series TLE499x [222], reach sensitivities up to 300 mV/mT at an operation voltage of 5.5 V and an operation range of ±200 mT. These high values are achieved through of the use of integrated amplifier circuits and enhance the intrinsic Hall effect in silicon. Such established integration technology is still missing for graphene, but improvements may be expected as the technology matures [13, 14]. Recent results indicate that graphene mobilities can be quite high when encapsulating graphene by Al_2_O_3_ [218], hBN [18, 46], and WSe_2_ [223]. This may be a promising route to also improve the performance of Hall sensors based on nonsuspended graphene [224, 225], which may be preferred for most applications, as it removes some of the fabrication challenges of suspended graphene membranes [146]. As discussed, the Hall effect provides an accurate method to detect the carrier concentration *n*. Suspended graphene Hall sensors, where the membrane is exposed to the environment, are thus promising as gas sensors, where molecules adsorbed to the graphene change its doping (=carrier density). Such sensors could be sensitive down to the single-molecule level [1].

## 11. Gas Sensors

### 11.1. Resistive Gas Sensors

2D material gas sensors can be used for environmental monitoring [12]. These are generally based on the adsorption of analytes such as NH_3_, CO_2_, H_2_O, and NO_2_ on the sensor surface [1, 150, 226–228]. This is in contrast to conventional metal oxide gas sensors based on zinc oxide (ZnO) or tin oxide (SnO_2_) that utilize surface reactions between oxygen and analyte molecules at grain boundaries. In 2D material gas sensors, the absorbed gas molecules induce charge carriers that cause an electrical resistance change in the sensor (chemiresistor) (Figures 7(g), 7(h), and 7(k)). Graphene chemiresistors are among the most investigated structures due to their simple fabrication, characterization, and miniaturization [150, 229–234], as well as potential use for biosensors [235]. In a so-called chemical field effect transistor (ChemFET) [1, 236, 237], the channel carrier concentration and conductance are modulated by applying a gate voltage to optimize gas sensing performance. Single-layer graphene and 2D materials have the substantial advantage of an inherent large surface area-to-volume ratio, but can also exhibit low Johnson-Nyquist noise [1] and 1/*f*_noise_ [238, 239]. This unique combination can result in very high signal-to-noise ratios and potentially lower detection limits towards the individual gas molecule level. Suspending the channel effectively doubles the available surface area and thus the achievable responsivity. In contrast, commercial chemiresistive gas sensors use, e.g., metal-oxide sensor materials, because they are very sensitive to multiple gases, but require high operation temperatures of 150°C [240], which are not needed in 2D material-based chemiresistive gas sensors. Also, the measurable concentration range of commercial gas sensors is limited, because they saturate at high gas concentrations [240]. This limitation is less evident in 2D materials [241]. 2D materials have been demonstrated with relative changes in resistance at room temperature of 39% at 200 ppm NO_2_ in air for graphene [242], 10% at 100 ppm NO_2_ in N_2_ for MoS_2_ [243], and 0.25% at 1 ppm NO_2_ in N_2_ for PtSe_2_ [119]. Suspended bilayer graphene was used to measure CO_2_ with high sensitivity (Figure 7(f)) [244]. MEMS MOS gas sensors based on silicon CMOS technology show resistivity changes from a few percent up to almost 100% for different target gases, but at operating temperatures of 300°C [245]. This results in high-power consumption of the sensors and thus limits their suitability for low-power applications such as smartphones.

Unfunctionalized suspended graphene resistors can also be used as gas sensors by measuring the thermal conductivity of a gas. A promising approach for improving response time and recovery time of indoor air quality sensors was demonstrated in [246], where resistive graphene-oxide humidity sensors have been suspended on MEMS micro hotplates and characterized using a temperature modulation procedure. Schottky barrier diodes have been demonstrated to be extremely sensitive gas sensors, in which the Schottky barrier height (SBH) depends on analyte exposure, which in turn modulates electrical currents. Kim et al. [247] proposed the effect of doping by liquid aromatic molecules on the SBH and Schottky diode ideality factor and Singh et al. demonstrated SBH modulation leading to a wide tunability of gaseous molecular detection sensitivity [248].

Although graphene gas sensors can be very sensitive, a challenge is to make them selective, since they often respond to many different gases and other parameters, which is similar to metal oxide sensors. Selectivity can be achieved through dedicated functionalization layers that enhance the reactivity only for certain gases. In addition to graphene, 2D materials such as MoS_2_ [112, 249], molybdenum diselenide (MoSe_2_) [110, 250], molybdenum ditelluride (MoTe_2_) [251], tungsten diselenide (WS_2_) [117], niobium diselenide (NbS_2_) [252], rhenium disulfide (ReS_2_) [253], or platinum diselenide (PtSe_2_) [119] have been shown to possess high gas and chemical sensor performance. Some TMD materials even show quite specific sensing behavior; in particular, PtSe_2_ has been shown to have a high selectivity towards NO_2_, which also was validated theoretically [119]. This may be exploited to enhance the sensitivity and selectivity through combining individual TMD sensors into sensor arrays [254]. Such sensor arrays, functionalized or unfunctionalized, can then be combined into an electronic nose [255]. Again, suspending these sensors will enhance the surface area and sensitivity, albeit at the cost of more challenging fabrication schemes, so that one has to choose an optimum cost/performance scenario.

Finally, repeatability and drift of gas sensors are a major general challenge, since the chemical binding energy of the gas molecules to the 2D material needs to be paid to remove the molecules and restore the sensor to its initial state. If the binding energy is close to *k*_*B*_*T*, this might be performed by heating; otherwise, light can be used to decrease recovery times.

### 11.2. Permeation-Based Gas Sensing

During the last decade, several works have demonstrated the feasibility of fast molecular sieving in gases and liquids using membranes made of 2D materials [256–258]. It was shown that pores with sub-1 nm diameters in these membranes can selectively sieve molecules or ions based on their molecular kinetic diameter. Specifically, it was shown [256] that small molecules such as H_2_ and CO_2_ permeate the membranes by a factor 1000 faster than argon, nitrogen, and methane gas. This methodology can also be used for permeation-based gas sensing, as was shown in [259] where a change in gas composition caused an osmotic pressure across a graphene membrane. This pressure is a consequence of the permeability differences of the different gases that effectively resulted in the graphene acting as a semipermeable membrane. For even larger pore sizes, when going from molecular sieving to effusion-dominated permeation, these sensing principles can be utilized for gas sensing [260], although with lower selectivity.

## 12. Graphene Mass Sensors

The low mass of graphene makes it an interesting candidate for accurate mass sensing. Such a sensor, shown in Figures 7(l)–7(o), determines a mass change of the membrane or ribbon by monitoring changes in its resonance frequency. The mass change can be introduced by adsorbed or attached atoms or molecules on the surface of the membrane. The responsivity of resonant mass sensors is given by Δ*ω*_res_ = −½*ω*_res_ Δ*m*/*m*_eff_ [261, 262], which shows that for a small mass *m* of the graphene membrane or ribbon, a relatively large frequency shift will occur. The high sensitivity of this principle was shown by adding and removing layers of pentacene with an equivalent mass of 6 layers of monolayer graphene and monitoring its effect on the resonance frequency of a graphene membrane (Figure 7(p)) [128]. Such suspended graphene resonant mass sensors are expected to find applications in fields where it is required to determine mass changes much less than a monolayer of a 2D material. In comparison, conventional quartz crystal monitors have been shown to be able to measure the mass of a single monolayer of graphene [128]. The sensitivity of graphene-based mass sensors can reach a value of 10^−27^ g/Hz [263], which greatly outperforms silicon membrane-based sensors, with typical sensitivity values of only 10^−18^ g/Hz [264]. Commercial mass sensors have even lower sensitivity values of around 60 × 10^−9^ g/Hz [265]. In the ultimate limit, graphene nanomembranes with diameters of below 10 nm, which often occur naturally in graphene on silicon oxide substrate, have been theoretically predicted to be able to detect one hydrogen atom of mass, which would lead to a relative resonance frequency shift of 10^−4^.

## 13. Graphene Bolometers

Bolometers are devices to detect absorption of electromagnetic radiation and light by monitoring the resulting temperature changes in a material via changes in its electrical resistivity. Especially for long wavelength infrared and THz radiation, bolometers are of interest, since there are few alternative detectors available in this frequency regime. At room temperature, where superconducting bolometers cannot be realized, suspended graphene is an interesting material for utilization of low-cost bolometers due to its ultra-wideband electromagnetic absorption and low heat capacitance due to its atomic thickness (Figures 7(q)–7(u)). The high thermal conductivity and low temperature coefficient of resistance of graphene are drawbacks that have recently been mitigated by instead utilizing a resonant readout mechanism in a focused ion-beam structured suspended graphene bolometer (Figure 7(q)) [266]. However, cross-sensitivity to other signals (e.g., thermoelectric and photoelectric) needs to be also dealt with. Graphene-based resonant radiation detectors for the infrared range show a noise equivalent power of about 2 pW/Hz at room temperature [266] and are thus in the upper range of conventional infrared bolometers based on vanadium oxide or nickel (1-10 pW/Hz) [267–271].

There are many other types of 2D material-based photosensors, but they are usually not suspended and fall therefore outside the scope of this review.

## 14. Discussion and Conclusions

While the field of silicon-based MEMS sensors is getting mature, the advent and discovery of 2D materials have brought us a set of nanomaterials for realizing novel NEMS sensors. Not only are these new materials thinner than any currently available CMOS or MEMS material, allowing drastic reductions of device size and enhanced sensitivity, there is also a larger range of materials emerging with exceptional properties. This large range of available material properties increases the freedom to engineer desired sensor properties for a particular application and to maximize sensitivity and reduce dimensions of the NEMS sensors. Moreover, by creating heterostructures of 2D materials, an even larger number of parameters will become available to optimize the sensor's electrical, mechanical, thermal, optical, chemical, and magnetic properties. The possibilities are expanding even further, since new types of ultrathin materials for NEMS applications continue to emerge, like those based on complex oxides [272] and 2D organic magnetic membranes [273].

In this review, we have given an overview of the NEMS sensors and proof-of-concept devices based on suspended 2D materials that have been demonstrated during the ast decade. These devices are almost always smaller than their conventional MEMS counterparts. Moreover, they show improved performance and sometimes even completely novel functionalities. Despite these successes, there are still enormous challenges ahead to demonstrate that 2D material-based NEMS sensors can outperform conventional devices on all important aspects. One of these tasks is the establishment of high-yield manufacturing capabilities [15]. We have given an overview and comparison of the different potential fabrication routes and their challenges, focusing on the challenges related to suspended sensors. In this respect, the recent EU experimental pilot line is expected to set a big step towards high quality, high-volume graphene devices [274]. Of course, a platform approach where multiple types of suspended sensors can be produced in a single production flow is desirable, but it remains to be seen to what extent this can be realized. Other remaining tasks are sensitive and customized electronic sensor readout circuits, packaging, and reliability testing for the 2D material NEMS sensors.

We believe that of all potential electronics applications for 2D materials, sensors made from nonsuspended 2D materials could be one of the first to become commercially available. Suspending the materials inherently adds process complexity and challenges and hence will likely take a longer time. Nevertheless, we are optimistic that, with joint efforts from both academia and industry, the first NEMS sensors based on 2D materials could hit the markets before the start of the next decade. In addition, 2D materials are now discussed for ultimate CMOS logic as stacked nanosheet transistors. This may trigger enormous, game-changing investments by industry that would upend any predictions made by us today.

## Figures and Tables

**Table 1:** Comparison of the most relevant properties of suspended 2D materials. Reported results are obtained from experiments on suspended membranes as well as 2D materials on various substrates.

	Young's modulus (GPa)	Poisson's ratio	Fracture strain (%)	Mobility (cm^2^/Vs)	Piezoresistive gauge factor	Optical bandgap (eV)
Highest-quality exfoliated graphene	800-1100 [17, 38]	0.11-0.2 [39–42]	0.3-30 [17, 42]	200000 (suspended) [43]	2-6 [32–34]	No bandgap
CVD polycrystalline graphene	1000 [44]	0.13-0.2 [39–41]	2 [45]	350000 (supported) [46]	2-6 [32–34]	No bandgap
h-BN	223 ± 16 [47]	0.21 [48]	17 [49]	Dielectric	—	5.9 [50]
MoS_2_	270 ± 100 [51]	0.27 [52]	6-11 [53]	73 (supported) [54]	−148 ± 19 (monolayer) [31] −224 ± 19 (bilayer) [31]	1.9 (monolayer) 1-1.6 (multilayer) [55, 56]
MoSe_2_	177.2 [57]	0.23 [57]	2.55 [57]	—	1800 (theory) [58]	1.59 [59]
PtSe_2_	—	—	—	Mostly <15; 210 [60]	Up to −85 ± 23 (few layer) [6]	1.2-1.6 (monolayer) 0.2-0.8 (bilayer) None (multilayer) [61, 62]
WS_2_	272 [63]	0.21 [64]	—	214 [65]	14 [37]	2 [66]
WSe_2_	167.3 [67]	0.19 [64]	7.3 [67]	—	3000 (theory) [58]	—
Black phosphorus	46-276 [68]	0.4 [68]	8-17 [68]	10000 (supported) [69]	69-460 [70, 71]	—
